# Efficacy of repetitive transcranial magnetic stimulation in Parkinson’s disease: an updated systematic review and meta-analysis of randomized controlled trials

**DOI:** 10.1097/MS9.0000000000003520

**Published:** 2025-07-09

**Authors:** Mahnoor Khattak, Mah Rukh, Warda Javed, Mujahid, Maryam Iftikhar, Najla Mumtaz, Ittehad Ul Mulk, Shifa Haleem, Sumaira Iram, Qaisar Ali Khan, Ravina Verma

**Affiliations:** a Department of Medicine, Khyber Teaching Hospital MTI KTH, Peshawar, Pakistan; b Department of Medicine, Lady Reading Hospital Peshawar, Pakistan; c Department of Medicine, Hayatabad Medical Complex Peshawar, Pakistan; d Department of Pediatric Emergency, Sultan Qaboos University, Oman; e Department of Surgery, St. George’s University School of Medicine True Blue, Grenada

**Keywords:** noninvasive brain stimulation, Parkinson’s disease, TMS, transcranial magnetic stimulation

## Abstract

**Background:**

Repetitive transcranial magnetic stimulation (rTMS) has emerged as a potential noninvasive treatment option for managing motor and non-motor symptoms in Parkinson’s disease (PD). While numerous randomized controlled trials have evaluated its efficacy, the evidence remains inconsistent. This updated systematic review and meta-analysis aims to consolidate the available data to assess the effectiveness of rTMS in improving motor symptoms, cognitive function, and quality of life in patients with PD.

**Method:**

A comprehensive search was conducted across multiple databases, including PubMed, Scopus, and Cochrane Library, from their inception to December 2024. Randomized controlled trials (RCTs) comparing rTMS with sham stimulation or other controls in patients with PD were included. The primary outcome was improved motor symptoms, as measured by the Unified Parkinson’s Disease Rating Scale (UPDRS) part III or MD-UPDRS-III. Data were pooled using a random effects model, and heterogeneity was assessed with the *I*^2^ statistic.

**Results:**

A total of 20 RCTs, including 693 participants, met the inclusion criteria. rTMS was associated with significant improvements in UPDRS-III (standardized mean difference [SMD] 0.53 [0.31, 0.74], *I*^2^ = 28%, *P* < 0.00001) and MD-UPDRS-III (SMD 0.42 [0.19, 0.66], *I*^2^ = 0%, *P* = 0.0005) scores. Cognitive function, assessed by Mini-Mental State Exam, showed no significant improvement. Beck Depression Inventory (SMD 0.36 [0.12, 0.61], *I*^2^ = 0%, *P* = 0.003) showed significant improvement. However, both HDRS (SMD 0.16 [−0.16, 0.47], *I*^2^ = 0%, *P* = 0.33) and MADRS (SMD 0.55 [−0.31, 1.41], *I*^2^ = 63%, *P* = 0.21).

**Conclusion:**

This updated meta-analysis provides evidence that rTMS is a safe and effective intervention for improving motor symptoms and cognitive function in PD patients. However, its effects on mood and long-term outcomes require further investigation.

## Introduction

Parkinson’s disease (PD) is a progressive neurodegenerative disorder marked by motor symptoms such as bradykinesia, tremors, and rigidity, alongside debilitating non-motor symptoms like depression and cognitive decline^[1]^. While dopaminergic therapies provide symptomatic relief, their long-term use often results in diminishing efficacy and motor complications, prompting the exploration of adjunctive interventions^[2]^.

Repetitive transcranial magnetic stimulation (rTMS), a noninvasive neuromodulation technique, has shown promise in addressing motor and non-motor symptoms of PD^[3]^. By modulating cortical excitability, particularly through high-frequency stimulation targeting the primary motor cortex (M1)^[4]^, rTMS improves motor function, as evidenced in several randomized controlled trials (RCTs)^[5,6]^. A 2022 meta-analysis by Zhang *et al*^[7]^ demonstrated moderate efficacy of rTMS for motor improvements and potential benefits for depressive symptoms, with notable variability based on stimulation parameters. Since then, the therapeutic landscape of PD has been broadened with an increasing number of studies exploring rTMS protocols and stimulation targets. These developments underscore the need for an updated analysis of evidence to determine whether these strategies offer additional clinical benefits over conventional approaches.

A recent study on cerebellar rTMS highlighted its potential as an alternative stimulation site for motor enhancement^[8]^. Additionally, integrating rTMS with EEG-guided neurofeedback demonstrated synergistic effects, significantly improving motor symptoms and quality of life^[9]^. Despite these promising findings, the overall efficacy of rTMS, the influence of stimulation parameters, and its impact on motor and non-motor outcomes remain unaddressed. Hence, these advancements necessitate an updated synthesis of evidence to refine clinical recommendations and address critical gaps.

Accordingly, this updated meta-analysis aims to evaluate the efficacy of rTMS in PD, incorporating recently published RCTs and examining the role of innovative stimulation strategies in enhancing therapeutic outcomes. Furthermore, we performed a meta-regression analysis to elucidate the effect of confounders such as male sex, disease duration, mean Hoehn and Yahr Scale stage (H&Y stage), and rTMS frequency on the primary outcome of motor function.

## Methodology

The systematic review and meta-analysis were conducted according to the Preferred Reporting Items for Systematic Reviews and Meta-Analyses (PRISMA), Assessing the Methodological Quality of Systematic Reviews, and the Transparency in the Reporting of Artificial Intelligence guidelines^[10,11]^.

### Literature search

A comprehensive literature search was performed using the electronic databases of PubMed, Scopus, and the Cochrane Library from inception to December 2024 for RCTs on rTMS intervention in PD. The search keywords were “Parkinson OR Parkinson’s disease OR PD” AND “transcranial magnetic stimulation OR TMS OR noninvasive brain stimulation.” Additionally, bibliographies of relevant articles were also searched to add additional articles. Conference proceedings and clinicaltrials.gov were queried to search for grey literature. The detailed search strategy is shown in the supplementary material (Supplemental Digital Content Table S1, available at: http://links.lww.com/MS9/A865).

### Eligibility criteria and quality assessment

RCTs were chosen to assess the efficacy of rTMS intervention on either motor or non-motor improvement in patients with PD. The following eligibility criteria were established: (A) English language articles were included, (B) patients were adults (>18 years of age), (C) motor function was measured by utilizing the motor section of the Unified Parkinson’s Disease Rating Scale (UPDRS Part III, a.k.a. UPDRS-III) and the motor section of Movement Disorder Society-Sponsored Revision of the Unified Parkinson’s Disease Rating Scale, (D) cognitive improvement and the antidepressant-like effect was included in non-motor function; cognitive improvement was measured by the Mini-Mental State Exam (MMSE) and the antidepressant-like effect was measured by the Beck Depression Inventory (BDI), Hamilton Depression Rating Scale (abbreviated as HDRS in this article, and sometimes abbreviated as HRSD and HAMD in other articles) or Montgomery − Asberg Depression Rating Scale (MADRS), (E) the trials presented their data in the form of continuous outcomes using means and standard deviations. Studies that had insufficient data, used noninvasive brain stimulation methods, utilized evaluation methods other than MD-UPDRS-III and UPDRS-III, or used combination therapy were excluded from the analysis. A quality assessment was performed using the risk of bias-2 tool of the Cochrane Collaboration^[11]^ to evaluate the RCTs’ validity and internal reliability. Studies were assessed for random sequence generation, allocation concealment, blinding of participants and personnel, blinding of outcome assessment, incomplete outcome data, and selective reporting.

**HIGHLIGHTS**

- Repetitive transcranial magnetic stimulation (rTMS) has emerged as a potential noninvasive treatment option for managing motor and non-motor symptoms in Parkinson’s disease.
- This updated meta-analysis aims to evaluate the efficacy of rTMS in Parkinson’s disease (PD), incorporating recently published randomized controlled trials and examining the role of innovative stimulation strategies in enhancing therapeutic outcomes.
- We performed a meta-regression analysis to elucidate the effect of confounders such as male sex, disease duration, mean Hoehn and Yahr Scale stage, and rTMS frequency on the primary outcome of motor function.
- This meta-analysis reaffirms the efficacy of rTMS in improving motor function in patients with PD, particularly with high-frequency and bilateral stimulation.
- However, its role in addressing cognitive and depressive symptoms remains uncertain, warranting further investigation.
- These findings underscore the importance of individualized treatment approaches and the need for robust, standardized research to optimize rTMS as a therapeutic tool in PD management.

### Data extraction and analysis

Two reviewers independently conducted data extraction and queries were resolved by mutual discussion. Data from the trials was added to a Microsoft Excel sheet where the following variables were added^[1]^: study design^[2]^; demographic characteristics (including number of patients, sex, and age)^[3]^; mean and SD of the scores of the motor and non-motor scales; and^[4]^ rTMS parameters (frequency, intensity, sessions, and site). RCTs, with multiple intervention groups, were subjected to a discussion to narrow it down to one group to compare with the sham group. Review Manager Version 5.3 was used to perform the meta-analysis. The results were pooled as standardized mean difference (SMD) using a random effects model and calculated with their 95% confidence interval. The findings were visualized via forest plots. A *P* value lower than 0.05 was considered significant. Heterogeneity across the pooled studies was assessed using the Higgins *I*^2^ statistics^[12]^. A value of *I*^2^ = 25%–50% was considered as mild, *I*^2^ = 50%–75% as substantial, and *I*^2^ > 75% as significant heterogeneity. Studies with high heterogeneity were subjected to sensitivity analysis to observe the difference in the significance of the outcomes. A funnel plot diagram was also drafted to ascertain publication bias wherever applicable. Lastly, meta-regression, using OpenMeta [Analyst] (version 5.26.14), was conducted to evaluate the correlation of the primary outcome with confounders such as male sex, disease duration, mean H&Y stage, and rTMS frequency. These results were reported as coefficients (Coeff) and P-values.

## Results

**Figure 1.**
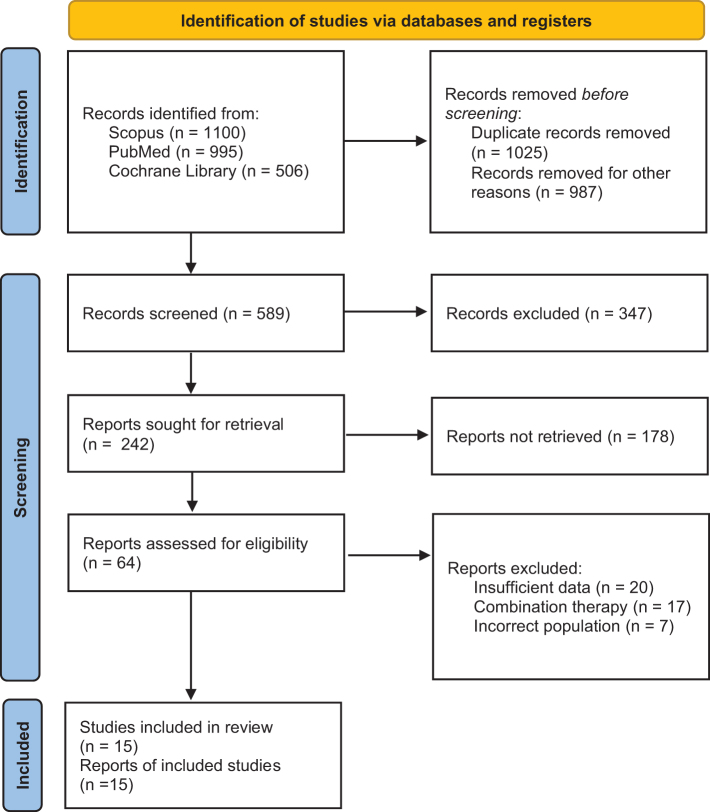
PRISMA flowchart.

The PRISMA screening flow diagram is shown in Figure 1. A total of 2601 articles were retrieved initially upon searching the databases. Duplicate records, non-RCTs, and studies in languages other than English were excluded. The remaining 589 records were screened thoroughly with articles removed that (a) had insufficient data, (b) used other types of noninvasive brain stimulation methods, (c) utilized evaluation methods other than MD-UPDRS-III and UPDRS-III, or (d) used any combination therapy. Finally, a total of 20 RCTs were included in the quantitative synthesis. The same study was eligible for inclusion in multiple assessments if it reported data on more than one stimulation site or frequency. Seventeen studies provided data on motor function, five on cognitive function, and nine on depression assessment.

### Patients characteristics

The 20 RCTs^[5,6,8,9,13–28]^ had a total of 693 patients with PD. The mean age across all included studies was 64 (9.4) years with the average disease duration being 7.5 (5.1) years. Five articles did not provide the Hoehn and Yahr Scale stage (H&Y stage) of the patients, and two did not provide disease duration. The detailed study and patient characteristics are included in Table 1.

**Table 1:** Study and patient characteristics of included trials

Study name	Sample size	Male sex	Mean age (SD)	Mean disease duration (SD)	Mean H&Y stage	rTMS site	rTMS frequency (Hz)
Filipovic 2009	10	5	64.5 (9.6)	15.6 (5.7)	NA	M1	1
Pal 2010	22	11	68.5 (7.9)	6.0 (4.8)	NA	DLPFC	5
Benninger 2012	26	20	64.1 (8.5)	9.0 (5.5)	2.6 (0.4)	M1	50
Marou 2013	21	11	63.0 (11.3)	12.0 (6.3)	3.1 (0.5)	M1	10
Kim 2015	17	12	64.5 (8.4)	7.8 (4.9)	3.0 (0.5)	M1	10
Makkos 2016	44	14	66.5 (8.0)	5.5 (4.8)	2.3 (0.8)	M1	5
Brys 2016	61	37	63.4 (10.0)	6.9 (4.7)	2.5 (0.6)	M1/DLPFC	10
Yokoe 2017	19	7	69.1 (8.4)	9.5 (3.2)	3.5 (0.6)	M1/DLPFC/SMA	10
Buard 2017	46	33	68.5 (7.6)	NA	NA	DLPFC	20
Cohen 2018	42	32	65.6 (7.5)	5.1 (3.5)	2.0 (0.37)	M1 + PFC	1 + 10
Khedr 2019	33	NA	59.5 (9.2)	6.0 (3.8)	3.2 (1.1)	M1	20
Mi 2019	30	14	63.6 (9.9)	8.6 (5.5)	2.5 (0.9)	SMA	10
Chung 2020	50	26	62.3 (6.0)	6.5 (4.0)	2.2 (0.3)	M1	1 + 25
Li 2020	48	32	61.6 (7.6)	6.0 (4.8)	1.8 (0.6)	M1	20
Spagnolo 2021	59	41	62.4 (8.1)	6.5 (1.9)	2.0 (0.2)	M1/DLPFC	10
Aftanas 2021	46	21	63.3 (7.8)	6.6 (4.1)	2.5 (0.6)	M1	10
Shaheen 2023	40	22	61.3 (6.8)	3.5 (2.2)	NA	M1	10
Barboza 2024	25	11	55.2 (9.0)	8.6 (7.5)	NA	PSI	10
Grobe-Einsler 2024	35	28	68.1 (10.2)	NA	1.97 (0.3)	Posterior cerebellum	48
Romero 2024	19	15	65.0 (8.3)	6.1 (3.5)	2.1 (0.5)	M1	10

### Assessment of motor function

A total of 17 studies were included in the evaluation of motor function following rTMS. Motor function was grouped based on the utilization of UPDRS-III or MD-UPDRS-III scales, frequency of rTMS, and type of stimulation.

### UPDRS-III or MD-UPDRS-III scales

**Figure 2.**
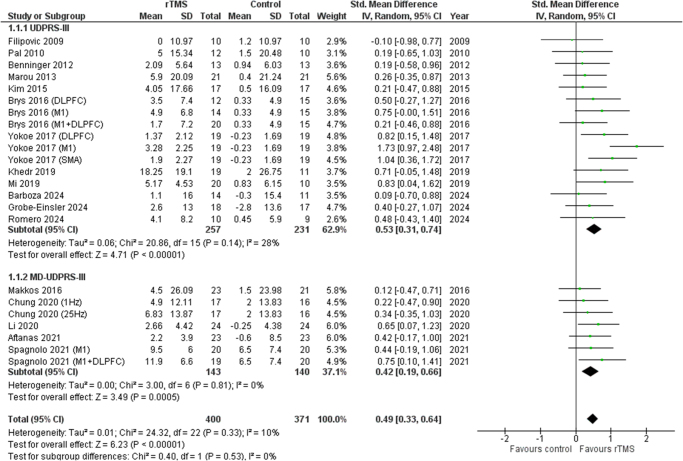
Forest plot of motor function.

A random-effects meta-analysis showed rTMS to be significantly efficacious in improving motor function when compared with the control group for both, UPDRS-III (SMD 0.53 [0.31, 0.74], *I*^2^ = 28%, *P* < 0.00001) and MD-UPDRS-III (SMD 0.42 [0.19, 0.66], *I*^2^ = 0%, *P* = 0.0005). Figure 2 shows the forest plot.

### Frequency of rTMS

**Figure 3.**
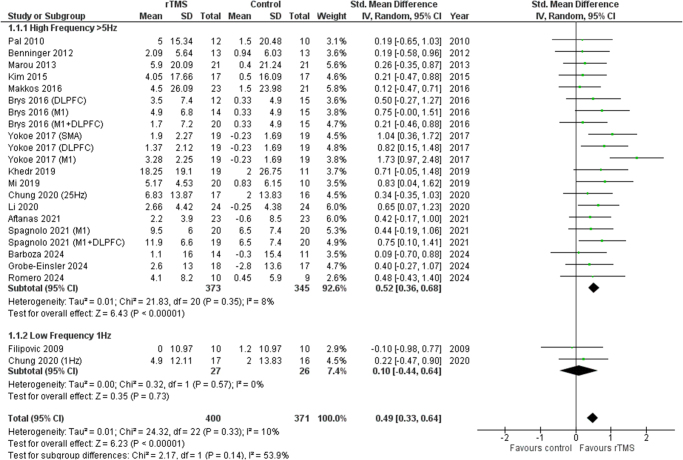
Forest plot of high vs low frequency rTMS.

High frequency was significantly associated with better motor function improvement (SMD 0.52 [0.36, 0.68], *I*^2^ = 8%, *P* < 0.00001) however, no significant association was seen with the low-frequency group (SMD 0.10 [−0.44, 0.64], *I*^2^ = 0%, *P* = 0.73). Figure 3 shows the forest plot.

### Type of stimulation

**Figure 4.**
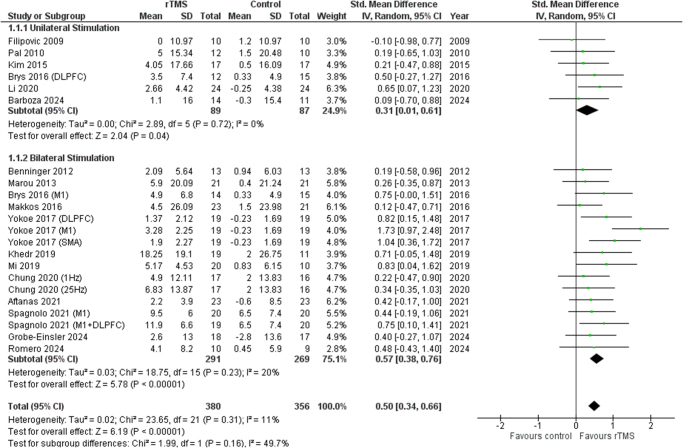
Forest plot of uni- and bilateral rTMS.

A significant improvement was ascertained with rTMS in both unilateral (SMD 0.31 [0.01, 0.61], *I*^2^ = 0%, *P* = 0.04) and bilateral stimulation (SMD 0.57 [0.38, 0.76], *I*^2^ = 20%, *P* = 0.73) when compared to the control group. Figure 4 shows the detailed result.

### Assessment of cognitive and depressive outcomes

**Figure 5.**
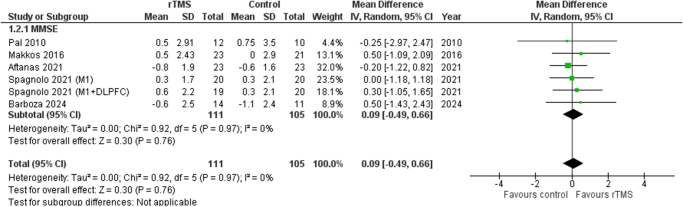
Forest plot of cognitive function.

MMSE was employed to evaluate improvement in cognition across the studies. A random-effects meta-analysis showed no significant association between rTMS and improvement in cognition following the stimulation (SMD 0.09 [−0.49, 0.66], *I*^2^ = 0%, *P* = 0.76). The forest plot is shown in Figure 5.

**Figure 6.**
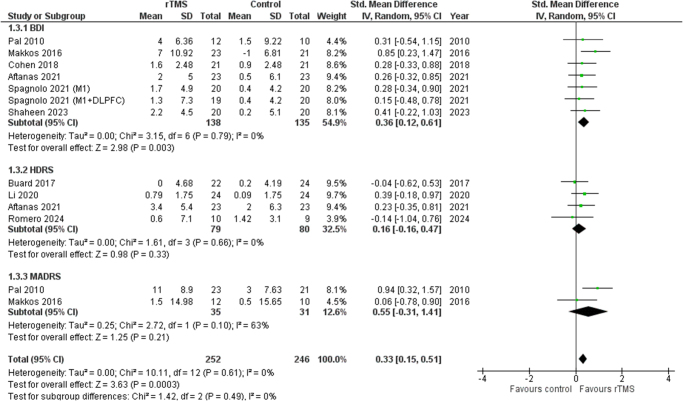
Forest plot of improvement in depression.

The outcome of depression was classified into BDI, HDRS, and MADRS, according to the scales. The BDI subgroup showed a significant improvement in depression following rTMS (SMD 0.36 [0.12, 0.61], *I*^2^ = 0%, *P* = 0.003). However, both HDRS (SMD 0.16 [−0.16, 0.47], *I*^2^ = 0%, *P* = 0.33) and MADRS (SMD 0.55 [−0.31, 1.41], *I*^2^ = 63%, *P* = 0.21) showed no significant association. Figure 6 shows the detailed forest plot.

### Quality assessment

RoB-2 tool was utilized to evaluate the quality of the included trials. All 20 RCTs were found to have a low risk of bias overall, however, some trials had some concerns in various domains (Supplemental Digital Content Figure S1, available at: http://links.lww.com/MS9/A859). Additionally, a funnel plot was generated to ascertain publication bias in the motor function outcome. A visible asymmetry was seen which could be predictive of publication bias (Supplemental Digital Content Figure S2, available at: http://links.lww.com/MS9/A860).

### Meta-regression

Male sex, disease duration, mean H&Y stage, and rTMS frequency were assessed as possible covariates having an impact on the primary outcome of assessment of motor symptoms. Only the mean H&Y stage was found to be a statistically significant predictor of improvement in motor symptoms following rTMS compared with the control group (Coeff: 0.2921, *P* = 0.0433) (Supplemental Digital Content Figure S5, available at: http://links.lww.com/MS9/A863). Other potential confounders had no significant association with motor function (Supplemental Digital Content Figure S3-S6 available at: http://links.lww.com/MS9/A889).

## Discussion

This updated meta-analysis comprehensively assesses the efficacy of rTMS in improving motor and non-motor outcomes in patients with PD. Based on 20 RCTs involving 693 patients, the findings indicate that rTMS significantly improves motor function, particularly with specific stimulation parameters such as high-frequency stimulation and bilateral application. However, its efficacy for cognitive and depressive symptoms appears to be more limited and variable, depending on the outcomes assessed.

The findings of our analysis validate the results obtained by Zhang *et al*^[7]^ which also demonstrated a significant improvement in motor symptoms following rTMS compared to conventional therapy. Similar to our results, Zhang *et al*^[7]^ reported that rTMS was significantly associated with overall improvement in depressive symptoms; however, their subgroup analysis indicated that this effect reached significance only when assessed using the BDI scale. Furthermore, similar to our findings, Zhang *et al*^[7]^ depicted no significant cognitive benefits with rTMS for PD.

The significant improvement in motor function, as measured by UPDRS-III and MD-UPDRS-III scales, highlights the potential therapeutic effect of rTMS as an intervention for PD. The effect size observed (SMD 0.53 and 0.42, respectively) is consistent with previous evidence^[7]^, suggesting its efficacy. These results are also in concordance with a meta-analysis conducted by Li *et al*^[29]^ that reported significant improvement in motor symptoms following rTMS therapy (SMD 0.64, 95% CI [0.47, 0.80]). The study found high-frequency stimulation, specifically, on M1, as the most effective method of intervention. The subgroup analysis of our study also highlights that high-frequency rTMS is particularly effective in improving motor symptoms, whereas low-frequency stimulation does not significantly benefit. This reaffirms the findings of Zhang *et al*^[7]^ which reported similar findings. This aligns with the neurophysiological mechanisms, wherein high-frequency stimulation facilitates cortical excitability, which may enhance motor circuit functionality in PD^[30]^.

The comparison of unilateral versus bilateral stimulation further highlights the importance of stimulation protocols. Both approaches showed significant improvements, however, the effect was more notable with bilateral stimulation (SMD 0.57 vs. 0.31). Bilateral stimulation may better address the widespread cortical dysfunction characteristic of PD, which might explain this finding. High-frequency rTMS facilitates motor learning by improving the interaction between M1 and SMA, leading to compensation by SMA for motor deficits in PD. High-frequency stimulation also has bilateral effects, even when applied unilaterally, due to transcallosal connections^[31]^. This explains why bilateral rTMS showed greater motor improvements in our study.

Despite the promising results for motor symptoms, the impact of rTMS on cognitive and depressive outcomes was less robust. Previous investigations have reported conflicting findings regarding the impact of rTMS on cognitive function in patients with PD^[32,33]^. However, these studies included small sample sizes and were not high-quality RCTs, prompting their results to be interpreted with caution. Jiang *et al*^[33]^ analyzed a total of 173 participants and found significant improvements in global cognitive functions following rTMS therapy. It should be noted however, that Jiang *et al*^[34]^ incorporated both RCTs and observational studies in their analysis. Similarly, Begemann *et al*^[35]^ demonstrated noninvasive brain stimulation to enhance working memory across various neurogenic disorders. The lack of significant improvement in cognitive function, as measured by MMSE, suggests that rTMS may not effectively target the neural networks implicated in cognitive decline in PD. Cognitive dysfunction in PD often involves widespread neurodegeneration beyond the cortical areas typically targeted by rTMS, which might account for this limited efficacy^[34]^. These findings were consistent with previous literature and rTMS has not been associated with an increase in cognitive function after stimulation^[7]^.

In terms of depression, the results were mixed. While a significant benefit was observed using the BDI scale (SMD 0.36), no improvement was noted with HDRS or MADRS scales. These discrepancies may stem from differences in the sensitivity of these scales to detect changes in depressive symptoms or variability in study designs and patient populations. Additionally, these results could be attributed to the lower number of studies in HDRS and MADRS. Furthermore, the heterogeneity observed in the MADRS subgroup (*I*^2^ = 63%) highlights the need for further exploration of optimal stimulation parameters for addressing depressive symptoms in PD. Several studies demonstrate the efficacy of rTMS in reducing depressive symptoms. Xie *et al*^[36]^ highlighted an improvement in depressive symptoms following rTMS therapy, and found a similar antidepressant efficacy in comparison to medications such as selective serotonin reuptake inhibitors. Similar findings were reported in another meta-analysis conducted by Lesenskyj *et al*^[37]^. Furthermore, evidence suggests that noninvasive brain stimulation is beneficial across several other depressive disorders. A study evaluated the impact of rTMS in the treatment of major depressive disorder and reported significant improvements^[38]^.

Lastly, upon conducting a meta-regression analysis, we concluded that studies involving patients with more severe PD (as represented by higher H&Y stage) showed increased motor improvement with rTMS as compared to control. A possible explanation for this finding is that patients with advanced PD may exhibit greater baseline dysfunction, allowing room for observable improvement following interventions like rTMS. Additionally, severe PD is accompanied by increased cortical plasticity and reorganization, allowing the brain to be more responsive to external stimulation^[39]^. Prior literature has observed greater responsiveness to noninvasive brain stimulation in patients with significant motor impairment^[40,41]^.

The findings of this meta-analysis underscore the clinical potential of rTMS as a noninvasive adjunctive therapy for motor symptoms in PD, particularly with high-frequency and bilateral stimulation protocols. However, the modest or nonsignificant effects on cognitive and depressive outcomes suggest the need for further research to refine rTMS protocols and explore combination therapies targeting these non-motor symptoms.

Future studies should aim to address the limitations of this meta-analysis, including separating the area of focus of stimulation and comparing the effects of high and low-frequency stimulation. Moreover, standardized reporting of rTMS parameters and consistent use of outcome measures would enhance the comparability and generalizability of findings. Investigating the long-term effects of rTMS and its impact on quality of life should also be prioritized in future research.

Several limitations should be acknowledged. First, the small sample sizes in many included studies may limit the meta-analysis’s statistical power. Second, variations in stimulation protocols, patient characteristics, and outcome measures contribute to heterogeneity, particularly for non-motor outcomes. Finally, the lack of data on long-term follow-up precludes conclusions regarding the sustainability of rTMS effects.

## Conclusion

This meta-analysis reaffirms the efficacy of rTMS in improving motor function in patients with PD, particularly with high-frequency and bilateral stimulation. However, its role in addressing cognitive and depressive symptoms remains uncertain, warranting further investigation. These findings underscore the importance of individualized treatment approaches and the need for robust, standardized research to optimize rTMS as a therapeutic tool in PD management.

## Data Availability

The datasets supporting the conclusions of this article are included within the article.

## Research registration unique identifying number (UIN)

PROSPERO, CRD42024591851, https://www.crd.york.ac.uk/prospero/#recordDetails.
